# Economic and health impacts of infectious diseases in China: A protocol for systematic review and meta analysis

**DOI:** 10.1097/MD.0000000000021249

**Published:** 2020-07-24

**Authors:** Meiyue Li, Danxue Fan, Xiaowen Wang

**Affiliations:** a School of Economics, Lanzhou University, Lanzhou; b Faculty of Economics and Management, Lanzhou Vocational Technical College, Lanzhou, China.

**Keywords:** Corona Virus Disease 2019, economic, influenza A, Avian Influenza Virus, health impact, meta-analysis, Severe Acute Respiratory Syndrome

## Abstract

**Background:**

A worldwide concern has been raised that novel infectious diseases may outbreak rapidly with a limited response time due to globalization. Severe Acute Respiratory Syndrome, influenza A, Avian Influenza Virus, and Corona Virus Disease 2019 are acute respiratory diseases that have been affected by the movements of people, and globalization accelerates these movements. These infectious diseases not only have an overwhelming health impact but also impact the worldwide economy.

**Methods:**

We will conduct a systematic review and meta-analysis in Chinese National Knowledge Infrastructure, WANFANG Database, and the VIP Database for Chinese Technical Periodicals, Web of Science, PubMed, EMBASE, the Cochrane Library, EBSCO host, ProQuest, ProQuest Dissertations & Theses A&I, SAGE Journals, ScienceDirect, JSTOR, and Scopus. We will evaluate the risk of bias of included RCTs according to the criteria and technique proposed in the Cochrane Handbook V.5.1.0 and use ROBINS-I to assess risk of bias in nonrandomized studies. We will use GRADE to evaluate the quality of evidence.

**Results:**

Results of this review will be submitted to a peer-reviewed journal.

**Conclusion:**

To the best of our knowledge, this study will firstly evaluate both health and economic impact of infectious diseases in china and may provide strategy development ideas for future resistance.

## 1 Introduction

With the rapid development of technology, international travelling increases the risk of outbreak of novel infectious disease. Respiratory infectious diseases mainly transmitted by near flying spittle and close-contact which may lead to fast transmission speed and high infection rate. People's lives are threatened when infectious diseases outbreak. Indeed, Infectious diseases cause a significant economic burden and a direct health impact.

China experienced several respiratory epidemics such as Severe Acute Respiratory Syndrome (SARS), influenza A (H1N1), Avian Influenza Virus (H7N9), and Corona Virus Disease 2019 (COVID-19) in the past decade. The SARS disaster in 2003 not only leads to serious consequences in China and other infected countries, but also provided precious experience for followed health emergencies. In 2003, SARS spread to 37 countries worldwide. In China, 20 provinces, regions and cities have detected infected cases.^[1]^ The pains from SARS accelerated the development of prevent and control infectious diseases systems in China. Stone suggest that China has taken stricter measures than other countries to defeat H1N1. ^[2]^ Then, China responded rapidly and transparently to H7N9 in 2013.^[3]^ Moreover, as of April 14, 2020, COVID-19 spread to 211 countries worldwide. Thirty-one provinces, regions, and cities in China reported infected cases.^[4]^ The response and control measures defeating COVID-19 has been taken quickly and effectively as well.

However, the economic and health impacts of these infectious diseases cannot be neglected. There are previous literatures focus on mortality burden of influenza and find that mortality depends on patients characteristics.^[5,6]^ Also, a previous systematic review and meta-analysis research the economic burden of influenza as well.^[7]^ Moreover, there is systematic review focus on the prevalence of human influenza in other countries.^[6,8–11]^ Pasquini–Descomps evaluates the cost-effectiveness to H1N1.^[12]^ Crawford et al reviews global health response to HIV/AIDS, SARS, H1N1, and Ebola.^[10]^ To the best of our knowledge, no previous systematic review or meta-analysis comprehensively studied both economic and health impact of infectious diseases in China. Besides, there is no systematic review summarized all papers discussed SARS, H1N1, H7N9, and COVID-19.

Hence, this protocol focused on both economic and health impact of infectious diseases in China. We especially focused on 4 infectious diseases which outbreak in China: SARS, H1N1, H7N9, and COVID-19. We expanded the inclusion criteria to include more related articles and also considered effectiveness of economic costs for infectious diseases.

## 2 Methods

### 2.1 Design and registration

In order to synthesise combinable research evidence, this protocol follows reporting items for systematic review and meta-analysis protocols.^[13]^ This protocol has been registered on PROSPERO (CRD42020173845).

### 2.2 Study eligibility criteria

#### 2.2.1 Type of study

This protocol includes randomized controlled trials (RCT), natural experiments and quasi-experimental study designs. There is no limitation on language and year of publication.

#### 2.2.2 Type of participants

Patients who acquired infectious diseases (including SARS, H1N1, H7N9 and COVID-19) in China are included. No restrictions were placed on gender, age, race, or nationality.

#### 2.2.3 Type of interventions

Certain types of infectious diseases are included. Studies focused on comparison between pre-infectious diseases time and post-infectious diseases time.

#### 2.2.4 Type of outcomes

The primary outcomes are economic cost, fiscal expenditure, mortality rate, and length of hospital stay. The secondary outcomes include Gross Domestic Product, Earning per share, and psychological status.

### 2.3 Selection of studies

This study will search articles comprehensively in Chinese National Knowledge Infrastructure, WANFANG Database, and the VIP Database for Chinese Technical Periodicals, Web of Science, PubMed, EMBASE, the Cochrane Library, EBSCO host, ProQuest, ProQuest Dissertations & Theses A&I, SAGE Journals, ScienceDirect, JSTOR, and Scopus. No restrictions were applied in language and publication period. Besides, this study will search unpublished papers, bibliographies of included papers manually. Search terms are as followed: health impact, economic impact, infectious diseases. The search strategy is as follows:

- #1 infectious disease OR infection OR infect OR infected OR infectious OR epidemic OR influenza OR virus OR pandemic OR outbreak SARS OR Severe Acute Respiratory Syndrome OR flu OR influenza A (H1N1) OR H1N1 OR avian influenza virus OR H7N9 OR COVID-19 Coronavirus Infections OR Coronavirus OR COVID-19 OR novel coronavirus OR SARS-CoV-2 OR 2019-nCoV
- #2 attributable cost OR economic burden OR economic OR Econom∗ factor OR Econom∗ determinant OR Econom∗ growth OR econom∗ development OR macroeconom∗ OR macro econom∗ OR Economics
- #3 mortality OR length of stay OR cost OR outcome OR health impact OR impact
- #4 #1 AND #2 AND #3

### 2.4 Data extraction

This study will use Endnote X7 (Clarivate Analytics, London, United Kingdom www.clarivate.com) to manage retrieved records. Fan and Li will screen the title and abstract independently. Then, we will access the full articles of selected articles and screened for further assessment. The disagreements between those 2 authors will be discussed by a third author. The data will be extracted from selected articles by 2 independent reviewers and formed using Microsoft Excel 2016. The details include descriptive characteristic (title, author, year of publication, published journal, region of research, analytical method, type of infectious diseases, data source, year of sample, and short description of findings) population characteristics (number of participates, participates characteristic) and outcome variables.

### 2.5 Risk of bias

Two reviewers will evaluate the risk of bias of included RCTs according to the criteria and technique proposed in the Cochrane Handbook V.5.1.0,^[14]^ which including random sequence generation, allocation concealment (selection bias), blinding of participants and personnel, blinding of outcome assessment, incomplete outcome data, selective reporting, and other bias.

The risk of bias will be evaluated according to the tool for assessing risk of bias in nonrandomized studies of interventions (ROBINS-I),^[15]^ including bias due to confounding, bias in selection of participants into the study, bias in classification of intervention, bias due to deviations from intended interventions, bias due to missing data, bias in measurement of outcomes, bias in selection of the reported result, and overall risk of bias. We will evaluate methodological quality as low, moderate, high risk of bias and no information.

### 2.6 Evidence quality

The Grades of Recommendation, Assessment, Development and Evaluation (GRADE) approach will be used to assess the quality of evidence for the main outcomes.^[16]^ According to GRADE, quality of evidence classified into 4 levels: high, moderate, low, and very low. There are 5 factors (risk of bias, indirectness, inconsistency, publication bias, and inaccuracy) may degrade the quality of evidence and 3 factors may improve the quality of evidence. The conflict will be discussed by an independent author.

### 2.7 Data synthesis

We will use Excel 2016 to summarize the data of all the included studies and manage their characteristics and data related to this systematic review and meta-analysis. This study will use Stata 15.1 (StataCorp, TX, USA, www.stata.com) to analyze the economic and health impacts of infectious diseases in China. Odds ratios (ORs) and Standardized mean difference (SMD) will be used as effect size in this study. Then, this study will report the d-value and the Confidence interval for effect size (95% CI) of included studies. The heterogeneity of each meta-analysis is tested by I^2^ statistics. A low *P* value of χ^2^ test and a high I^2^ value represent heterogeneity.

### 2.8 Subgroup and publication bias

For considerable heterogeneity studies, this study will adopt subgroup analysis and sensitivity analysis. Considering the publication bias, this study will draw a funnel plot by using Begg's and Egger's methods as well.^[17,18]^

## Author contributions

ML, DF and XW planned and designed the research; DF and ML tested the feasibility of the study; ML wrote the manuscript; all authors approved the final version of the manuscript.

**Conceptualization:** M. Li, D. Fan, X. Wang.

**Data curation:** M. Li, D. Fan.

**Investigation:** M. Li, D. Fan, X. Wang.

**Methodology:** M. Li, D. Fan.

**Supervision:** X. Wang.

**Writing – original draft:** M. Li.

**Writing – review & editing:** M. Li, D. Fan, X. Wang.
